# Supplementary material for: Effect of the Nanorough Surface of TiO2 Thin Films on the Compatibility with Endothelial Cells
Source: Int J Mol Sci. 2023 Apr 3;24(7):6699. doi: 10.3390/ijms24076699 (PMC10095362; doi:10.3390/ijms24076699)
Supplement: Supplementary file 1 [file ijms-24-06699-s001.zip › ijms-2281530-supplementary.pdf]

Supporting information

Effect of the Nanorough Surface of TiO_2 Thin Films on the Compatibility with Endothelial Cells

Irina Yu. Zhuravleva, Maria A. Surovtseva, Andrey A.Vaver, Evgeny A. Suprun, Irina I. Kim, Natalia A. Bondarenko, Oleg S. Kuzmin, Alexander P. Mayorov and Olga V. Poveshchenko

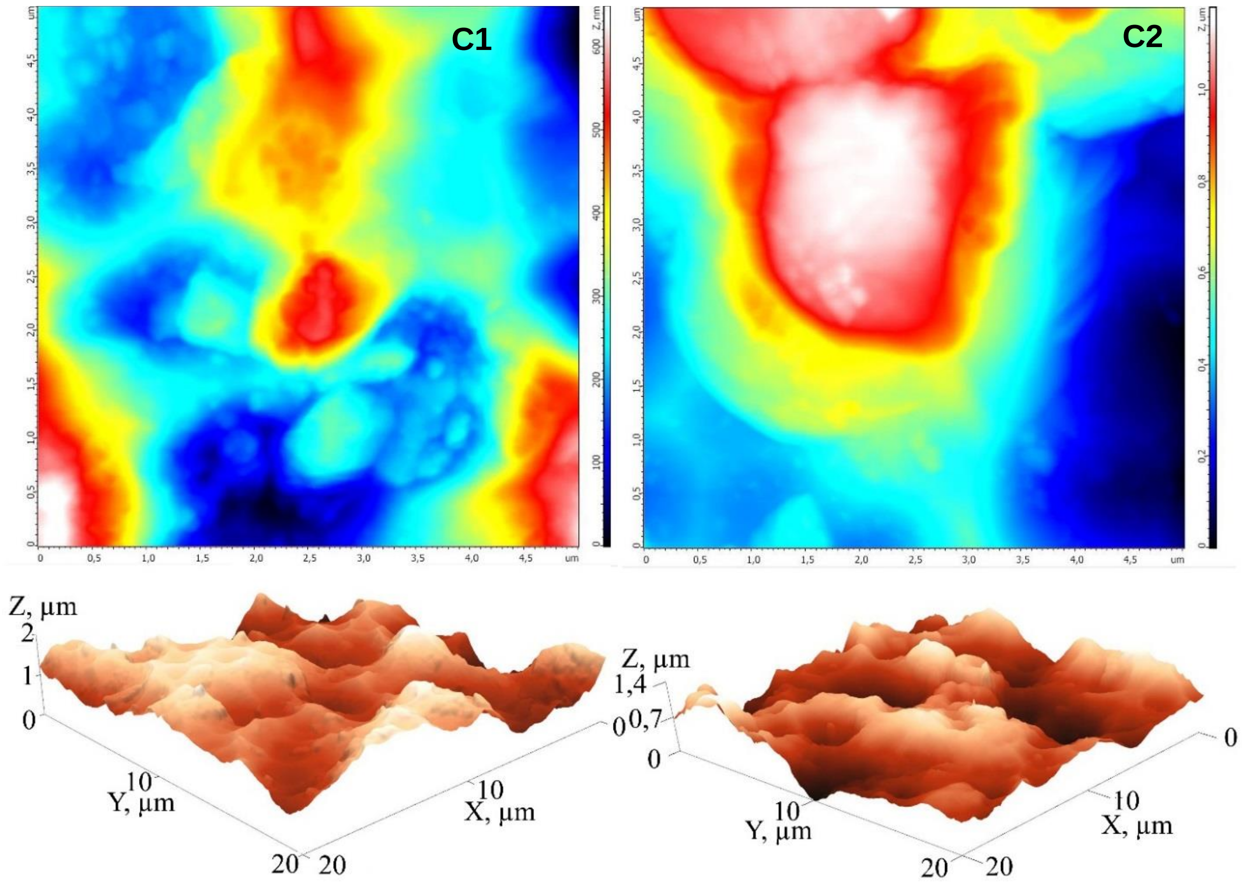

Figure S1. AFM images of control sample surfaces. C1 nitinol samples were machine polished, C2 samples were abraded by hand.

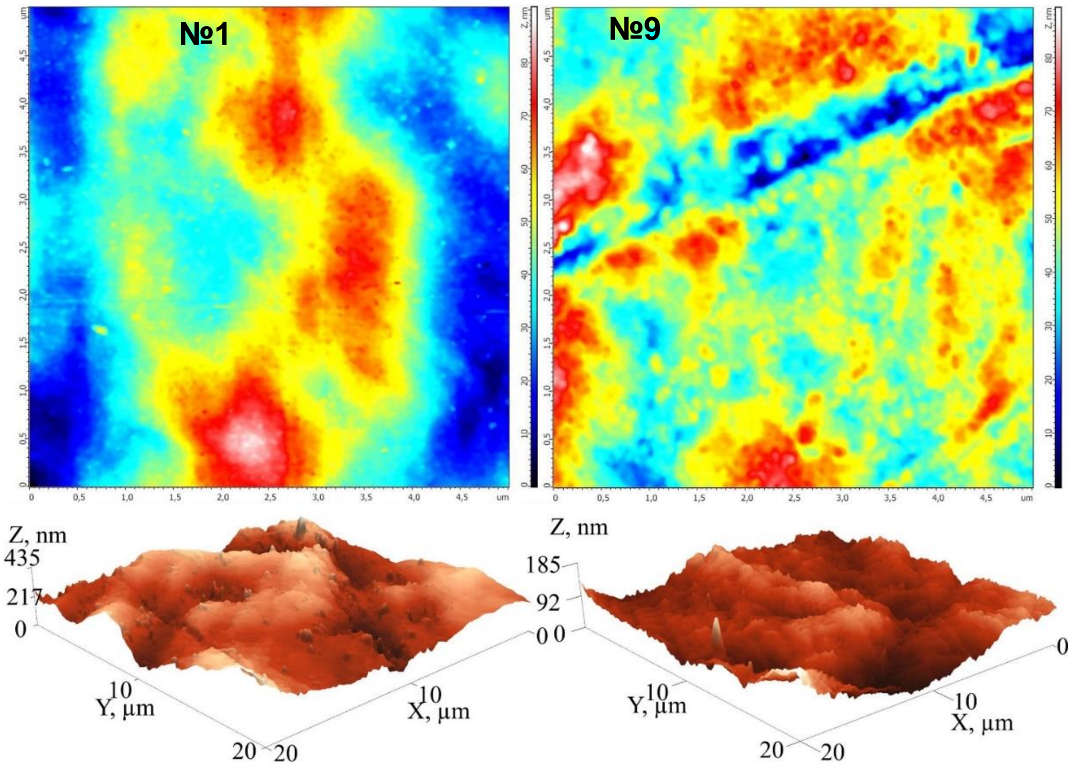

Figure S2. AFM images of samples coated with TiO_2 thin films. Magnetron sputtering at U_{bias}= -100 V. NiTi substrate were abraded by hand (№1) or machine polished (№9).

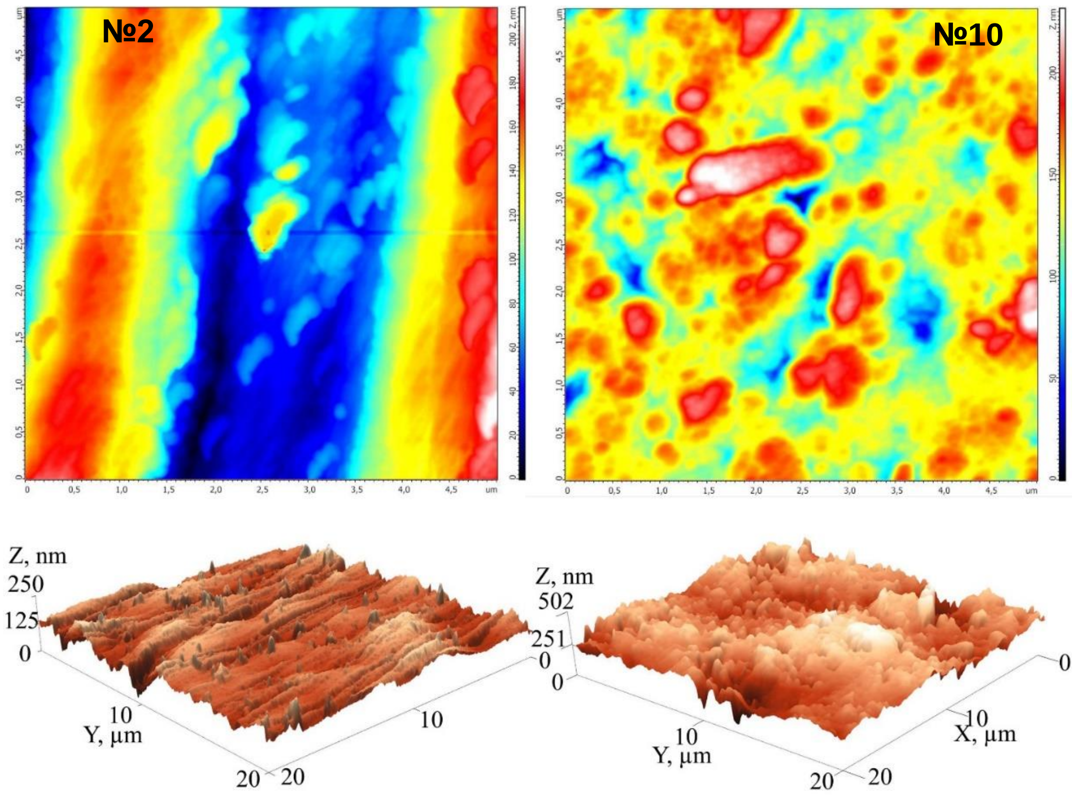

Figure S3. AFM images of samples coated with TiO_2 thin films. Magnetron sputtering at U_{bias}= 0 V. NiTi substrate were abraded by hand (№2) or machine polished (№10).

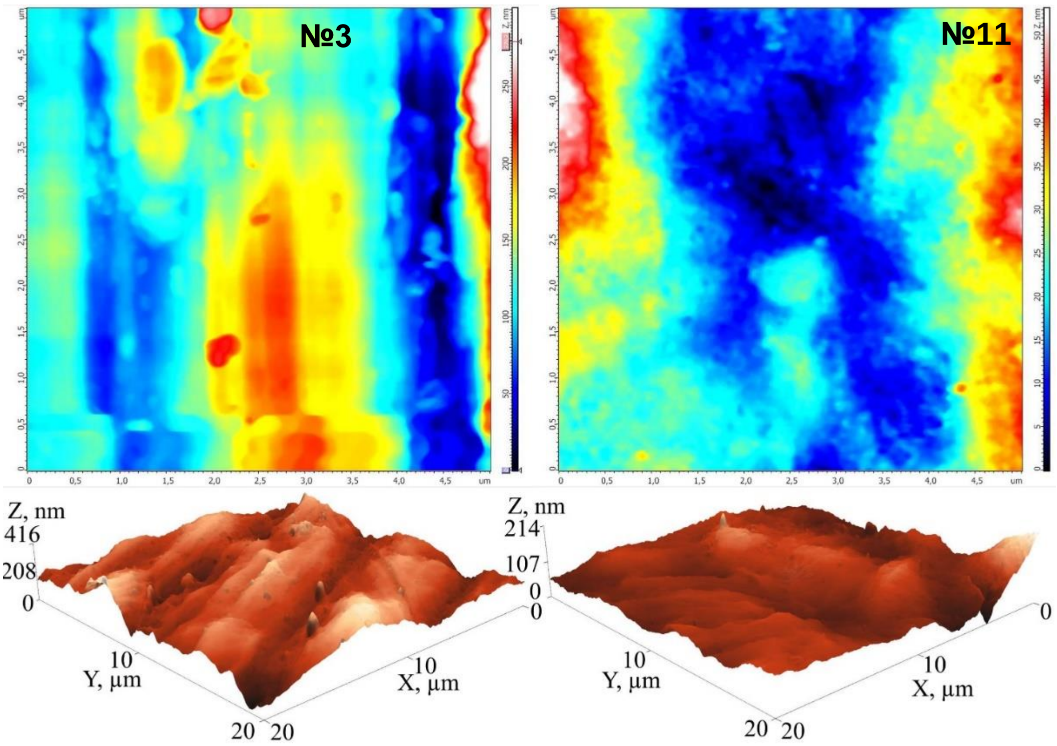

Figure S4. AFM images of samples coated with TiO_2 thin films at gas ratio $O_2:N_2=1:1$. Magnetron sputtering at U_{bias}= -100 V. NiTi substrate were abraded by hand (№3) or machine polished (№11).

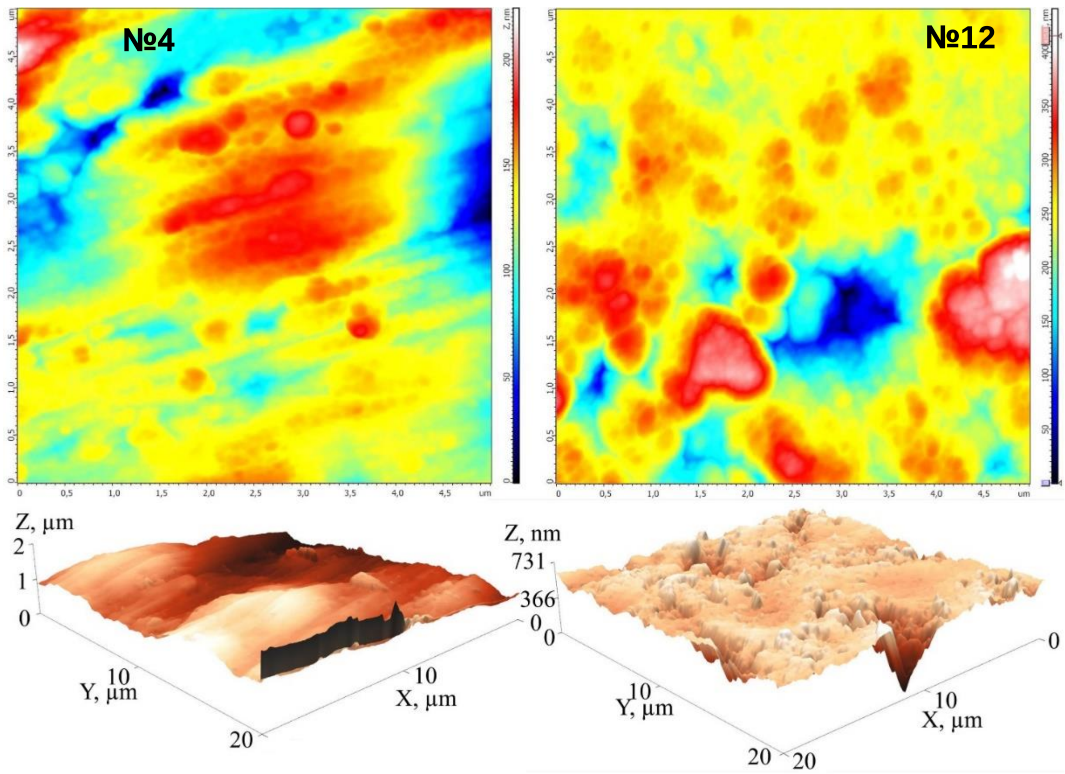

Figure S5. AFM images of samples coated with TiO_2 thin films at gas ratio $O_2:N_2=1:1$. Magnetron sputtering at U_{bias}= 0 V. NiTi substrate were abraded by hand (№4) or machine polished (№12).

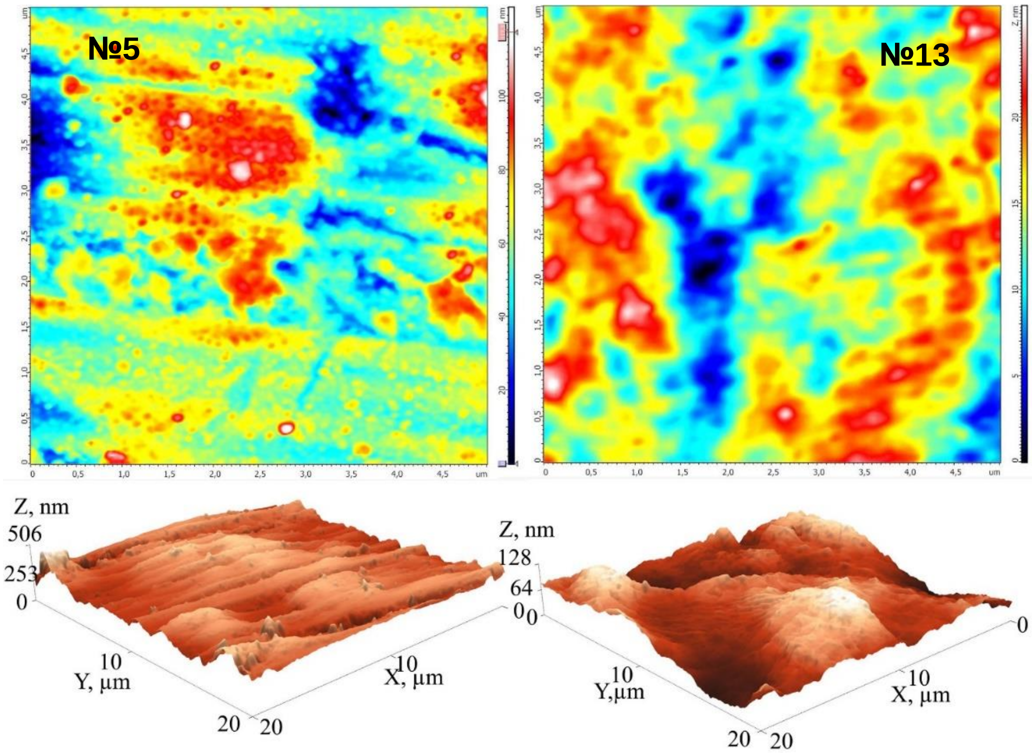

Figure S6. AFM images of samples coated with TiO_2 thin films at gas ratio O_2:N_2=1:2. Magnetron sputtering at U_{bias}= -100 V. NiTi substrate were abraded by hand (№5) or machine polished (№13).

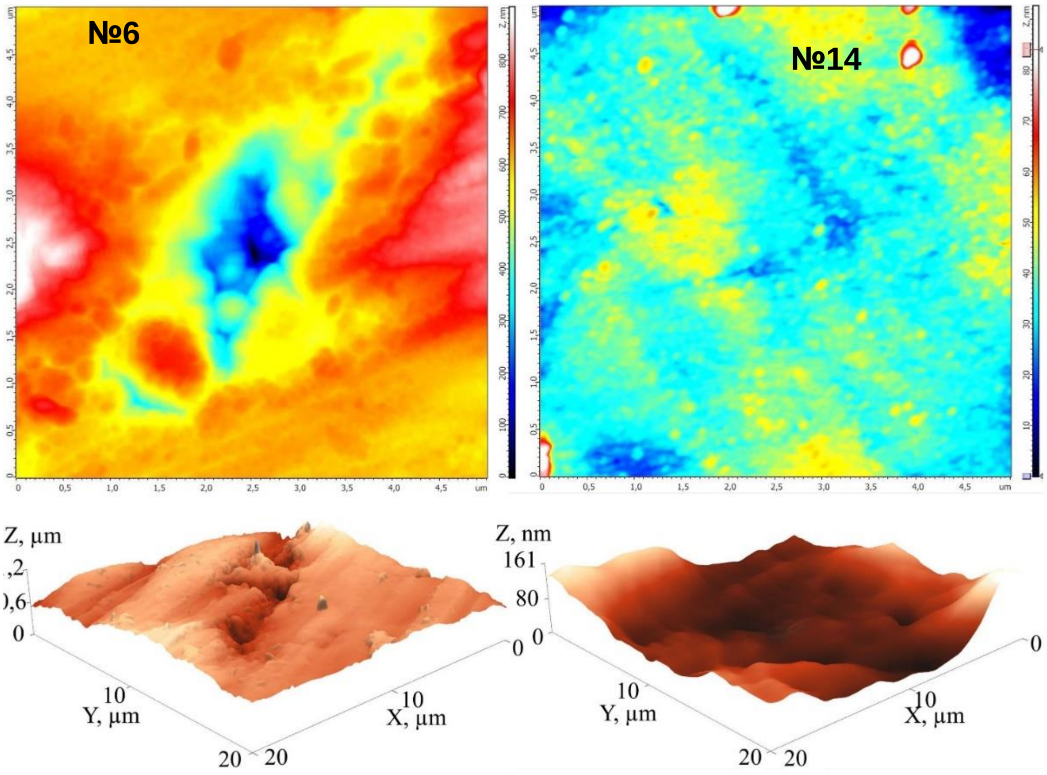

Figure S7. AFM images of samples coated with TiO_2 thin films at gas ratio O_2:N_2=1:2. Magnetron sputtering at U_{bias}= 0 V. NiTi substrate were abraded by hand (№6) or machine polished (№14).

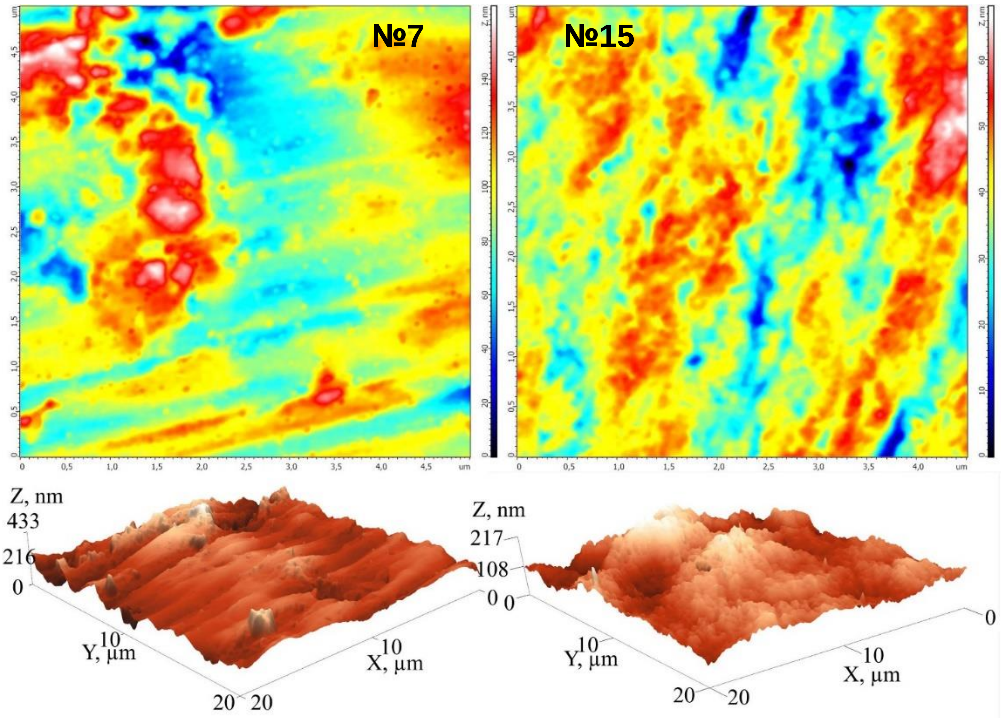

Figure S8. AFM images of samples coated with TiO_2 thin films at gas ratio O_2:N_2=1:3. Magnetron sputtering at U_{bias}= -100 V. NiTi substrate were abraded by hand (№7) or machine polished (№15).

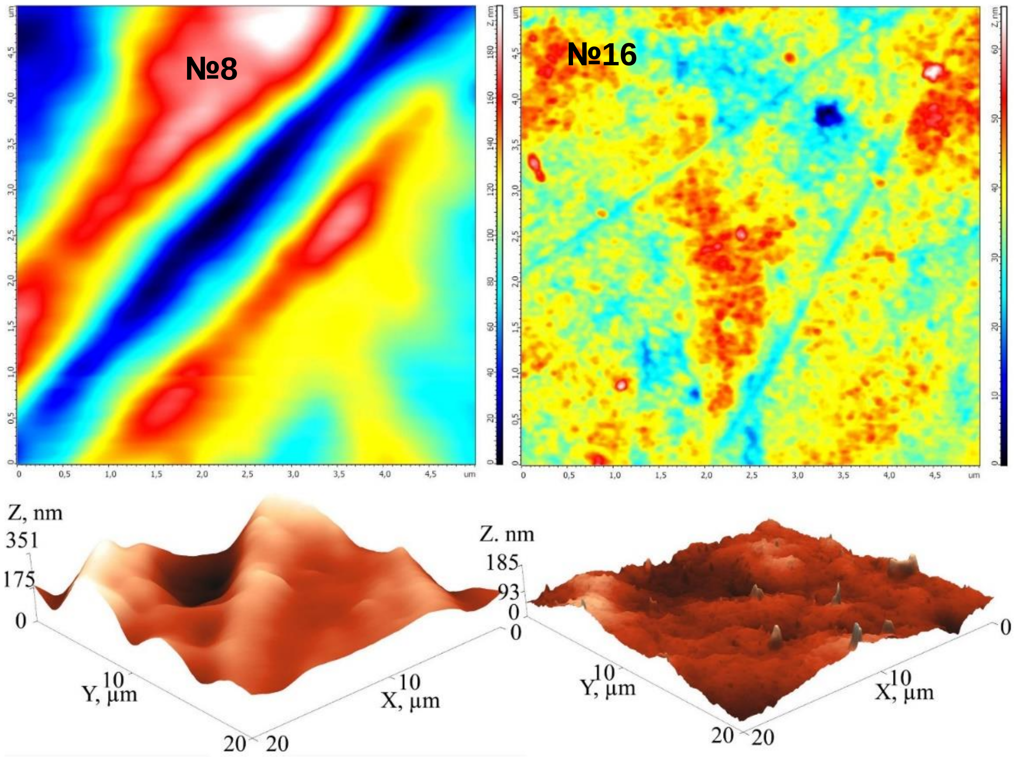

Figure S9. AFM images of samples coated with TiO_2 thin films at gas ratio O_2:N_2=1:3. Magnetron sputtering at U_{bias}= 0 V. NiTi substrate were abraded by hand (№8) or machine polished (№16).

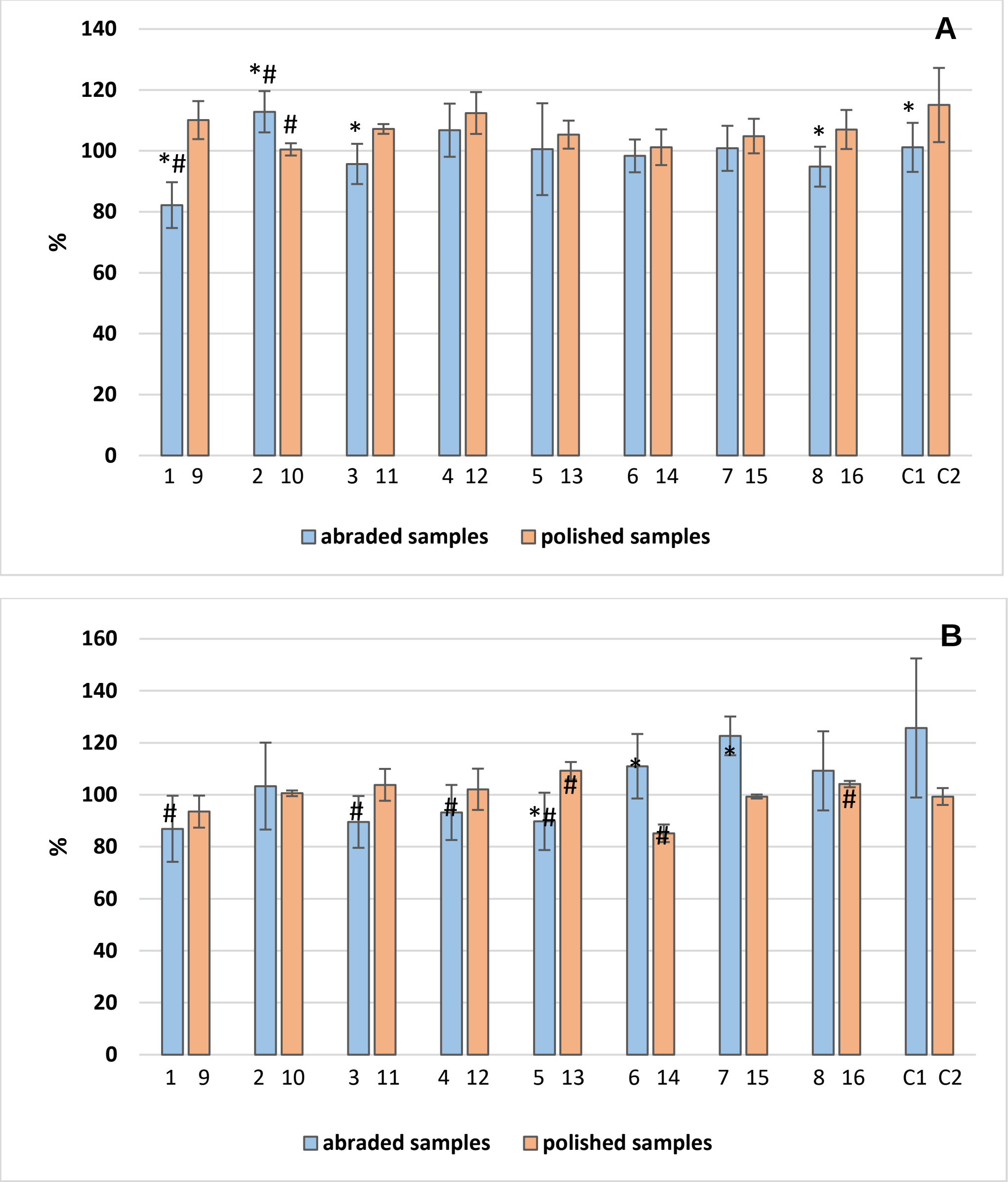

Figure S10. The viabilities of endothelial cells exposed to sample extracts during 24 h (A) and 72h (B); control group (100%) was cells cultivated in the DMEM/F12 medium without added extracts.

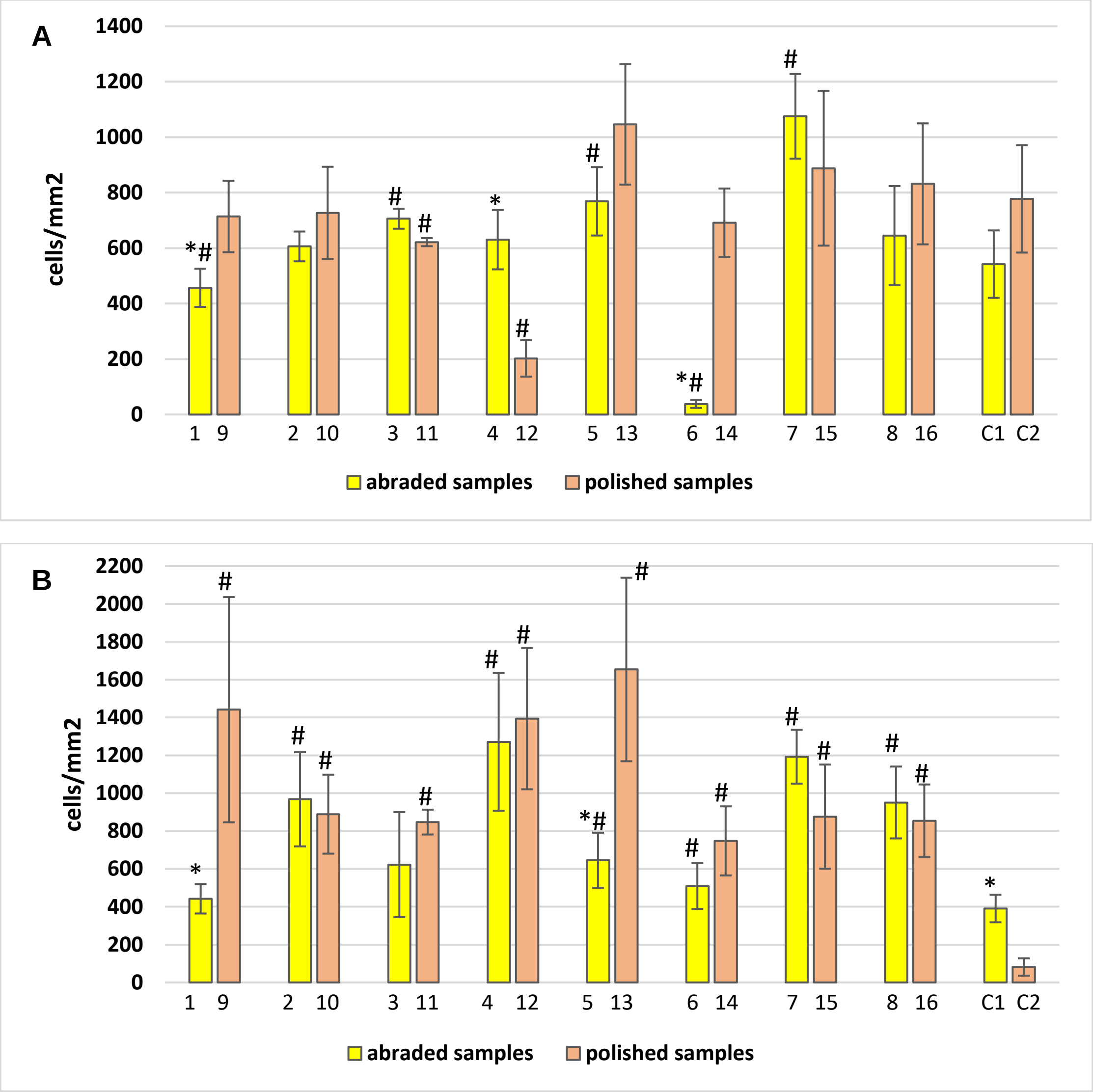

Figure S11. EA.hy926 cells’ adhesion on the sample surfaces after 3 (A) and 6 (B) days.

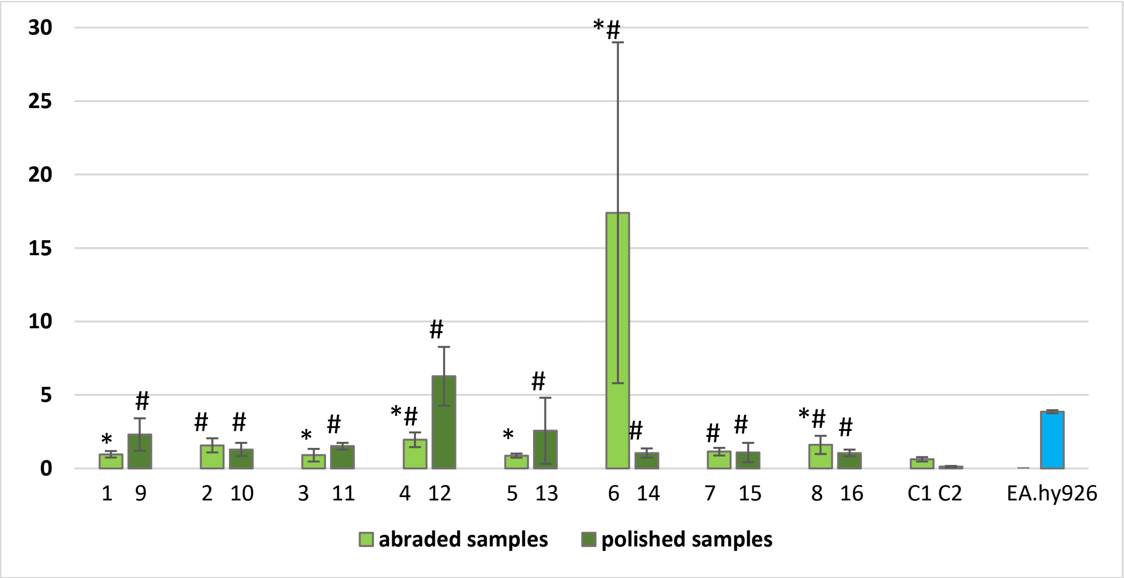

Figure S12. Proliferation index of EA.hy926 cells on abraded and polished surface of samples

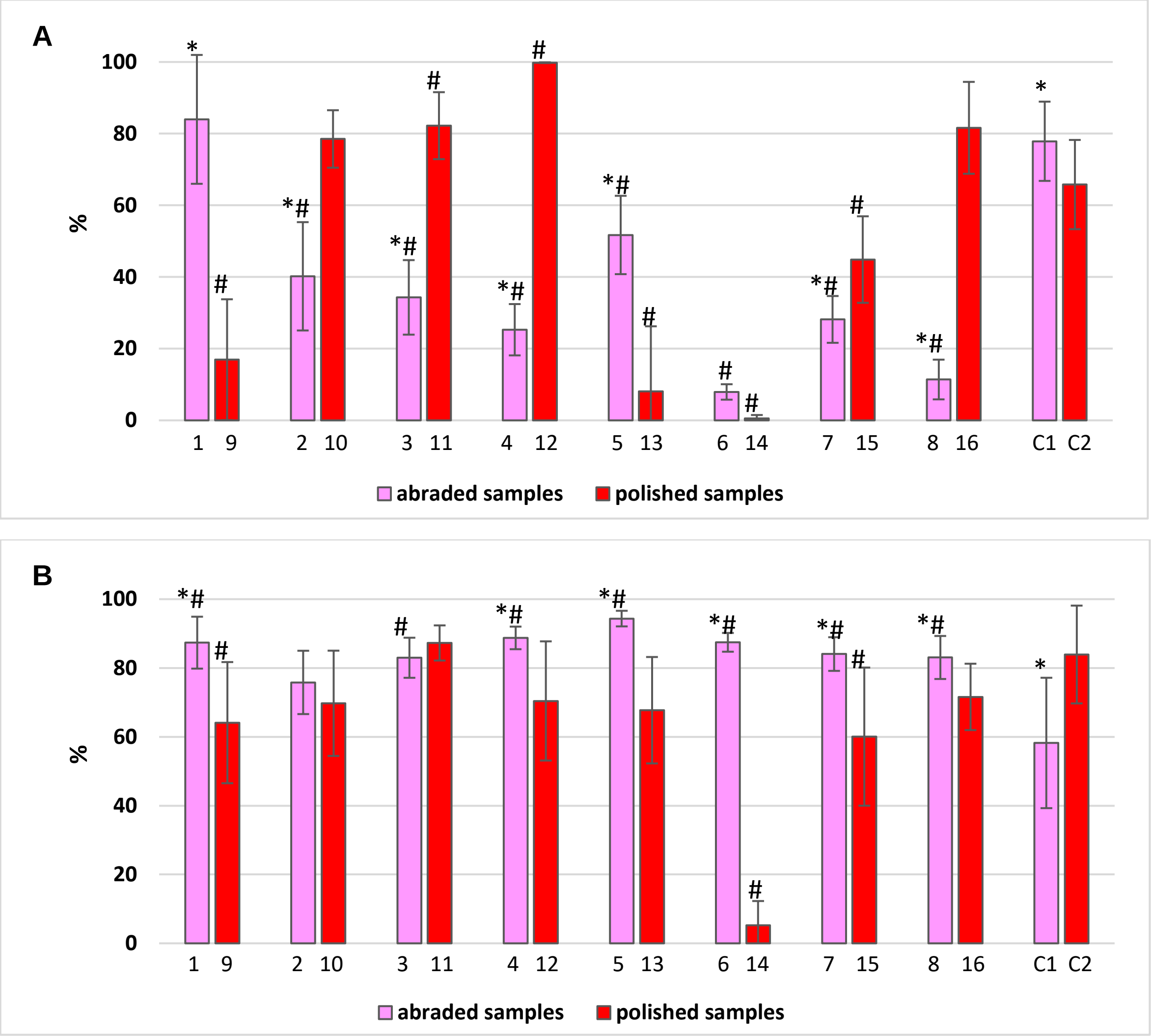

Figure S13. Viability of the EA.hy926 cells on abraded and polished surfaces samples after 3 (A) and 6 (B) days.

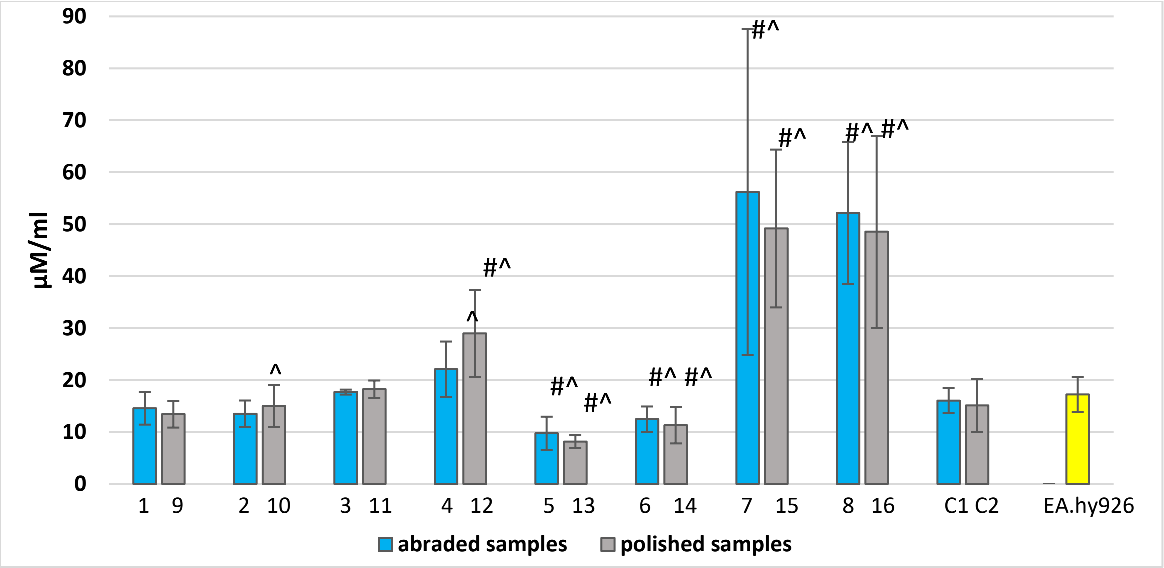

Figure S14. NO production of EA.hy926 cells on abraded and polished surfaces samples.

Table S1. Differences in Sq, Root mean square height of the surface

	02	1	2	3	4	5	6	7	8	9	10	11	12	13	14	15	16
01	0,008	0,406	0,118	0,011	0,005	0,007	0,003	0,435	0,097	0,626	0,845	0,770	0,205	0,242	0,448	0,914	0,329
02		0,002	0,001	0,001	0,001	0,001	0,001	0,001	0,001	0,006	0,001	0,001	0,002	0,001	0,001	0,002	0,002
1			1,000	0,025	0,003	0,022	0,035	0,848	0,565	0,277	0,406	0,655	0,025	0,655	0,668	0,465	0,291
2				0,055	0,006	0,010	0,018	0,406	0,949	0,085	0,110	0,180	0,013	0,565	0,253	0,223	0,042
3					0,035	0,775	0,749	0,035	0,142	0,003	0,004	0,009	0,002	0,064	0,007	0,007	0,004
4						0,317	0,406	0,004	0,035	0,002	0,002	0,002	0,002	0,006	0,003	0,004	0,004
5							1,000	0,010	0,086	0,003	0,003	0,010	0,003	0,032	0,004	0,006	0,006
6								0,009	0,085	0,004	0,002	0,002	0,002	0,035	0,003	0,012	0,004
7									0,482	0,142	0,482	0,482	0,009	0,749	1,000	0,465	0,062
8										0,025	0,085	0,085	0,002	0,565	0,253	0,123	0,012
9											0,338	0,277	0,110	0,048	0,199	0,465	0,465
10												0,949	0,004	0,180	0,253	0,935	0,123
11													0,006	0,180	0,475	0,935	0,123
12														0,004	0,003	0,012	0,061
13															0,475	0,223	0,012
14																0,523	0,045
15																	0,347

Table S2. Differences in maximum height of the surface (Sz)

	02	1	2	3	4	5	6	7	8	9	10	11	12	13	14	15	16
01	0,019	0,172	0,845	0,172	0,001	0,002	0,118	0,922	0,329	0,064	0,380	0,143	0,097	0,205	0,329	0,462	0,902
02		0,015	0,006	0,001	0,001	0,001	0,001	0,001	0,001	0,626	0,040	0,118	0,329	0,015	0,001	0,066	0,003
1			0,160	0,013	0,002	0,003	0,009	0,035	0,035	0,085	0,406	0,277	0,406	0,949	0,003	0,807	0,166
2				0,371	0,006	0,010	0,180	0,749	0,406	0,025	0,180	0,048	0,048	0,180	0,391	0,291	0,807
3					0,004	0,010	0,565	0,142	0,949	0,003	0,035	0,003	0,004	0,013	0,775	0,042	0,223
4						0,253	0,025	0,002	0,035	0,002	0,004	0,002	0,002	0,002	0,010	0,004	0,019
5							0,199	0,003	0,199	0,003	0,007	0,003	0,003	0,003	0,055	0,006	0,068
6								0,142	0,749	0,003	0,013	0,003	0,004	0,013	0,475	0,042	0,123
7									0,565	0,003	0,025	0,004	0,009	0,048	0,199	0,291	0,372
8										0,004	0,013	0,004	0,009	0,025	1,000	0,088	0,167
9											0,180	0,110	0,565	0,110	0,003	0,062	0,012
10												0,655	0,406	0,338	0,022	0,808	0,062
11													0,749	0,277	0,003	0,465	0,012
12														0,277	0,003	0,223	0,062
13															0,003	0,808	0,291
14																0,083	0,100
15																	0,347

Table S3. Differences in maximum peak height (Sp)

	02	1	2	3	4	5	6	7	8	9	10	11	12	13	14	15	16
01	0,290	0,922	0,329	0,380	0,001	0,007	0,079	0,495	0,172	0,380	0,922	0,770	0,845	0,495	1,000	0,713	0,540
02		0,079	0,019	0,008	0,001	0,001	0,006	0,019	0,011	0,696	0,205	0,097	0,380	0,032	0,129	0,221	0,086
1			0,125	0,064	0,002	0,003	0,063	0,277	0,110	0,035	0,749	0,949	0,749	0,406	0,668	0,935	0,807
2				0,798	0,004	0,032	0,406	0,655	0,749	0,018	0,277	0,142	0,225	0,655	0,199	0,570	0,291
3					0,004	0,007	0,338	0,655	0,655	0,002	0,110	0,035	0,225	0,749	0,032	0,167	0,088
4						0,116	0,018	0,002	0,018	0,002	0,009	0,002	0,003	0,002	0,003	0,012	0,028
5							0,253	0,004	0,063	0,003	0,022	0,004	0,010	0,007	0,004	0,045	0,100
6								0,225	0,848	0,003	0,085	0,064	0,085	0,180	0,046	0,167	0,291
7									0,565	0,003	0,180	0,085	0,337	0,949	0,022	0,167	0,123
8										0,003	0,142	0,064	0,110	0,406	0,046	0,167	0,291
9											0,064	0,048	0,482	0,035	0,022	0,088	0,028
10												0,848	0,749	0,406	0,886	0,935	0,808
11													0,655	0,277	0,886	0,570	0,685
12														0,338	0,775	0,685	0,685
13															0,253	0,465	0,685
14																0,648	1,000
15																	0,602

Table S4. Differences in kurtosis of height distribution (Sku)

	02	1	2	3	4	5	6	7	8	9	10	11	12	13	14	15	16
01	0,034	0,008	0,079	0,051	0,205	0,065	0,051	0,097	0,172	0,008	0,143	0,032	0,032	0,019	0,515	0,037	0,178
02		0,143	0,329	0,922	0,097	0,588	0,914	0,845	0,172	0,242	0,283	1,000	0,380	0,770	0,009	1,000	0,391
1			0,100	0,180	0,035	0,116	0,475	0,064	0,142	0,406	0,048	0,180	0,225	0,338	0,022	0,372	0,088
2				0,307	0,565	0,886	0,749	0,406	0,406	0,180	0,749	0,406	0,338	0,277	0,032	0,291	0,808
3					0,085	0,475	0,886	0,749	0,110	0,225	0,180	0,949	0,482	0,655	0,004	0,935	0,291
4						0,123	0,317	0,085	0,655	0,013	0,655	0,074	0,064	0,025	0,063	0,123	0,570
5							0,668	0,668	0,253	0,063	0,568	0,391	0,317	0,199	0,016	0,361	0,465
6								0,848	0,338	0,406	0,482	0,848	0,565	0,848	0,015	0,935	0,372
7									0,142	0,064	0,482	0,655	0,277	0,338	0,003	0,935	0,372
8										0,048	0,338	0,142	0,110	0,064	0,116	0,088	0,465
9											0,013	0,338	0,482	0,482	0,003	0,372	0,019
10												0,142	0,085	0,074	0,015	0,123	0,685
11													0,655	0,798	0,004	0,808	0,223
12														0,848	0,004	0,935	0,167
13															0,003	0,570	0,088
14																0,014	0,045
15																	0,076

Table S5. Differences in mean summit curvature (Ssc)

	02	1	2	3	4	5	6	7	8	9	10	11	12	13	14	15	16
01	0,002	0,283	0,006	0,283	0,001	0,001	0,770	0,015	0,017	0,845	0,001	0,845	0,733	0,015	0,001	0,020	0,002
02		0,097	0,001	0,118	0,001	0,001	0,097	0,001	0,001	0,040	0,011	0,040	0,040	0,001	0,001	0,002	0,002
1			0,007	0,142	0,002	0,003	0,338	0,013	0,004	0,142	0,004	0,180	0,406	0,002	0,003	0,004	0,004
2				1,000	0,002	0,063	0,085	0,654	0,180	0,013	0,002	0,013	0,009	0,225	0,003	0,372	0,004
3					0,110	0,617	0,565	0,749	0,949	0,406	0,004	0,277	0,338	0,749	0,474	0,570	0,684
4						0,022	0,025	0,002	0,025	0,002	0,002	0,002	0,002	0,003	0,022	0,088	0,004
5							0,032	0,063	0,032	0,003	0,003	0,003	0,003	0,022	0,078	0,855	0,067
6								0,179	0,277	0,338	0,013	0,701	0,482	0,482	0,032	0,291	0,042
7									0,565	0,013	0,003	0,025	0,018	0,749	0,015	0,371	0,028
8										0,035	0,002	0,048	0,015	0,848	0,032	0,570	0,042
9											0,004	0,749	0,749	0,047	0,003	0,042	0,004
10												0,004	0,006	0,002	0,003	0,004	0,004
11													0,565	0,064	0,003	0,062	0,004
12														0,035	0,003	0,028	0,004
13															0,022	0,372	0,042
14																1,000	0,582
15																	0,753

Table S6. Differences in density of summits of the surface (Sds)

	02	1	2	3	4	5	6	7	8	9	10	11	12	13	14	15	16
01	0,001	0,001	0,283	0,696	0,845	0,278	0,011	0,329	0,079	0,143	0,001	0,002	0,495	0,079	0,017	0,540	0,002
02		0,001	0,015	0,558	0,001	0,001	0,205	0,015	0,329	0,380	0,001	0,097	0,079	0,079	0,001	0,462	0,002
1			0,011	0,142	0,002	0,003	0,482	0,013	0,013	0,013	0,035	0,110	0,025	0,002	0,003	0,042	0,004
2				0,371	0,048	0,116	0,009	0,110	0,142	0,018	0,003	0,013	0,110	0,085	0,116	0,570	0,004
3					0,749	0,886	0,110	0,949	0,949	0,565	0,013	0,277	0,749	0,848	0,015	0,570	0,004
4						0,153	0,013	0,180	0,025	0,085	0,002	0,002	0,338	0,025	0,004	0,570	0,004
5							0,007	0,153	0,032	0,015	0,003	0,003	0,175	0,032	0,008	0,584	0,006
6								0,064	0,064	0,085	0,125	0,565	0,125	0,055	0,003	0,088	0,004
7									0,482	0,655	0,003	0,048	0,848	0,482	0,010	0,808	0,004
8										0,898	0,002	0,097	0,482	0,749	0,032	1,000	0,012
9											0,003	0,125	0,655	0,949	0,003	0,685	0,004
10												0,035	0,018	0,002	0,003	0,007	0,004
11													0,048	0,110	0,003	0,223	0,004
12														0,482	0,032	0,935	0,004
13															0,004	0,570	0,004
14																0,083	0,006
15																	0,006

Table S7. Statistical significance of differences in cell viabilities after 24-h incubation in sample extracts

	1	2	3	4	5	6	7	8	9	10	11	12	13	14	15
2	0.000														
3	0.013	0.002													
4	0.001	0.248	0.047												
5	0.035	0.127	0.526	0.442											
6	0.003	0.004	0.504	0.096	0.765										
7	0.003	0.023	0.274	0.272	0.970	0.557									
8	0.018	0.002	0.834	0.034	0.453	0.373	0.203								
9	0.005	0.575	0.013	0.378	0.230	0.013	0.066	0.005							
10	0.005	0.013	0.230	0.378	0.810	0.810	0.936	0.128	0.005						
11	0.005	0.230	0.013	0.575	0.378	0.005	0.128	0.005	1.000	0.005					
12	0.005	0.936	0.013	0.230	0.128	0.013	0.066	0.005	0.378	0.005	0.378				
13	0.005	0.128	0.031	0.936	0.575	0.066	0.230	0.031	0.378	0.031	0.810	0.128			
14	0.013	0.031	0.230	0.378	0.936	0.378	0.810	0.230	0.031	0.810	0.031	0.031	0.378		
15	0.013	0.128	0.031	0.936	0.575	0.066	0.378	0.031	0.378	0.378	0.810	0.128	0.810	0.378	
16	0.005	0.128	0.031	0.936	0.575	0.066	0.128	0.031	0.230	0.031	0.378	0.128	0.810	0.230	0.810

Table S8. Statistical significance of differences in cell viabilities after 72-h incubation in sample extracts

	1	2	3	4	5	6	7	8	9	10	11	12	13	14	15
2	0.080														
3	0.698	0.114													
4	0.375	0.238	0.554												
5	0.688	0.128	0.973	0.595											
6	0.008	0.391	0.008	0.024	0.011										
7	0.000	0.027	0.000	0.000	0.000	0.076									
8	0.020	0.540	0.025	0.061	0.030	0.830	0.080								
9	0.471	0.378	0.575	0.810	0.936	0.013	0.005	0.093							
10	0.066	0.378	0.031	0.230	0.066	0.066	0.005	0.378	0.378						
11	0.020	0.378	0.066	0.173	0.020	0.378	0.005	0.378	0.378	1.000					
12	0.031	0.936	0.066	0.378	0.066	0.378	0.013	0.575	0.378	0.810	1.000				
13	0.005	0.936	0.005	0.005	0.005	0.810	0.013	0.810	0.378	0.005	0.378	0.128			
14	0.575	0.230	0.575	0.378	0.378	0.005	0.005	0.013	0.031	0.005	0.005	0.005	0.005		
15	0.066	0.378	0.128	0.575	0.066	0.066	0.005	0.230	0.378	0.066	0.378	0.810	0.005	0.005	
16	0.066	0.378	0.005	0.013	0.013	0.230	0.005	0.378	0.378	0.005	1.000	0.378	0.005	0.005	0.005

Table S9. Statistical significance of differences in cell viabilities between 24-h and 72-h incubation in sample extracts

1	2	3	4	5	6	7	8	C1	9	10	11	12	13	14	15	16	C2
0.345	0.463	0.225	0.225	0.173	0.116	0.028	0.116	0.043	0.917	0.463	0.345	0.028	0.463	0.028	0.116	0.917	0.116

Table S10. Statistical significance of differences in cell adhesion after three-day cultivation on sample surfaces

	1	2	3	4	5	6	7	8	9	10	11	12	13	14	15
2	0.037														
3	0.037	0.037													
4	0.053	0.624	0.713												
5	0.020	0.066	0.713	0.241											
6	0.020	0.020	0.020	0.014	0.014										
7	0.020	0.020	0.020	0.014	0.043	0.014									
8	0.270	0.540	1.000	0.670	0.594	0.014	0.020								
9	0.014	0.171	0.855	0.411	0.784	0.008	0.014	0.784							
10	0.012	0.210	0.676	0.296	0.835	0.012	0.022	0.754	1.000						
11	0.036	0.648	0.144	0.784	0.083	0.008	0.008	0.648	0.036	0.076					
12	0.006	0.006	0.006	0.006	0.006	0.006	0.006	0.006	0.008	0.012	0.008				
13	0.083	0.083	0.121	0.121	0.171	0.008	0.927	0.171	0.235	0.403	0.171	0.006			
14	0.012	0.296	0.531	0.403	0.296	0.012	0.012	0.835	0.784	0.676	0.235	0.006	0.121		
15	0.008	0.055	0.523	0.083	0.523	0.008	0.523	0.315	0.315	0.403	0.055	0.006	0.784	0.296	
16	0.012	0.047	0.531	0.095	0.676	0.012	0.060	0.210	0.523	0.403	0.121	0.006	0.411	0.403	0.784

Table S11. Statistical significance of differences in cell adhesion after six-day cultivation on sample surfaces.

	1	2	3	4	5	6	7	8	9	10	11	12	13	14	15
2	0.004														
3	0.262	0.078													
4	0.004	0.262	0.016												
5	0.025	0.030	0.873	0.010											
6	0.128	0.004	0.262	0.004	0.037										
7	0.004	0.150	0.004	0.337	0.004	0.004									
8	0.004	1.000	0.016	0.150	0.010	0.004	0.025								
9	0.005	0.230	0.036	0.915	0.014	0.008	0.784	0.171							
10	0.008	0.784	0.210	0.111	0.095	0.012	0.060	0.403	0.121						
11	0.008	0.523	0.296	0.020	0.012	0.012	0.012	0.296	0.083	0.037					
12	0.008	0.036	0.012	0.391	0.012	0.012	0.403	0.037	0.784	0.022	0.012				
13	0.008	0.036	0.012	0.327	0.012	0.012	0.210	0.022	0.523	0.391	0.012	0.403			
14	0.043	0.337	0.540	0.030	0.270	0.178	0.020	0.178	0.043	0.676	0.713	0.020	0.020		
15	0.013	0.471	0.171	0.110	0.171	0.055	0.055	0.784	0.121	0.676	0.835	0.037	0.037	0.713	
16	0.005	0.522	0.315	0.070	0.068	0.008	0.036	0.235	0.083	0.676	1.000	0.037	0.022	1.000	0.855

Table S12. Statistical significance of differences in cell adhesion between three and six-day cultivation on sample surfaces

1	2	3	4	5	6	7	8	C1	9	10	11	12	13	14	15	16	C2
0.893	0.043	0.686	0.028	0.138	0.043	0.225	0.080	0.046	0.028	0.345	0.043	0.043	0.225	0.686	0.917	0.686	0.018

Table S13. Statistical significance of differences in cell viability after three-day cultivation on sample surfaces

	1	2	3	4	5	6	7	8	9	10	11	12	13	14	15
2	0.022														
3	0.012	0.403													
4	0.012	0.095	0.144												
5	0.037	0.210	0.060	0.014											
6	0.012	0.012	0.012	0.014	0.014										
7	0.012	0.144	0.210	0.594	0.014	0.014									
8	0.012	0.022	0.012	0.070	0.014	0.014	0.020								
9	0.008	0.083	0.121	0.173	0.013	0.066	0.171	0.784							
10	0.144	0.012	0.012	0.008	0.008	0.008	0.012	0.012	0.008						
11	0.315	0.008	0.008	0.005	0.005	0.005	0.008	0.008	0.005	0.648					
12	0.008	0.008	0.008	0.005	0.005	0.005	0.008	0.008	0.005	0.008	0.005				
13	0.008	0.036	0.055	0.066	0.020	0.378	0.083	0.083	0.378	0.008	0.005	0.006			
14	0.012	0.012	0.012	0.008	0.008	0.315	0.012	0.012	0.171	0.012	0.008	0.006	0.927		
15	0.036	0.648	0.235	0.013	0.378	0.005	0.036	0.008	0.022	0.012	0.008	0.006	0.022	0.012	
16	0.676	0.012	0.012	0.008	0.022	0.008	0.012	0.012	0.008	0.676	0.927	0.006	0.008	0.012	0.008

Table S14. Statistical significance of differences in cell viability after six-day cultivation on sample surfaces

	1	2	3	4	5	6	7	8	9	10	11	12	13	14	15
2	0.055														
3	0.262	0.150													
4	0.749	0.006	0.025												
5	0.109	0.004	0.004	0.006											
6	1.000	0.010	0.055	0.423	0.004										
7	0.423	0.109	0.873	0.055	0.004	0.200									
8	0.337	0.262	0.873	0.078	0.006	0.200	0.749								
9	0.031	0.378	0.121	0.022	0.008	0.036	0.083	0.055							
10	0.036	0.523	0.060	0.012	0.012	0.012	0.060	0.060	0.927						
11	0.784	0.036	0.403	0.676	0.060	1.000	0.531	0.296	0.036	0.022					
12	0.083	0.648	0.210	0.022	0.012	0.022	0.144	0.531	0.648	0.676	0.037				
13	0.055	0.235	0.144	0.144	0.022	0.144	0.144	0.144	0.784	0.531	0.095	0.835			
14	0.014	0.014	0.020	0.020	0.020	0.020	0.020	0.020	0.014	0.020	0.020	0.020	0.020		
15	0.008	0.173	0.022	0.008	0.008	0.008	0.014	0.036	0.411	0.144	0.012	0.210	0.927	0.014	
16	0.020	0.471	0.055	0.008	0.008	0.008	0.036	0.121	0.471	0.927	0.014	1.000	0.523	0.014	0.471

Table S15. Statistical significance of differences in cell viability between three and six-day cultivation on sample surfaces

1	2	3	4	5	6	7	8	C1	9	10	11	12	13	14	15	16	C2
0.686	0.043	0.043	0.043	0.043	0.043	0.043	0.043	0.007	0.028	0.500	0.138	0.043	0.043	0.068	0.249	0.225	0.176

Table S16. Statistical significance of differences in NO production over 24 h incubation on samples

	2	3	4	5	6	7	8	9	10	11	12	13	14	15	16
1	0.746	0.189	0.023	0.05	0.746	0.004	0.003	0.515	0.123	0.348	0.048	0.008	0.645	0.008	0.008
2		0.004	0.004	0.023	0.106	0.003	0.003	0.234	0.874	0.212	0.048	0.112	0.554	0.004	0.004
3			0.189	0.004	0.004	0.003	0.003	0.048	0.248	0.334	0.048	0.008	0.025	0.025	0.025
4				0.004	0.004	0.003	0.003	0.048	0.389	0.901	0.312	0.008	0.648	0.005	0.005
5					0.024	0.003	0.003	0.607	0.557	0.048	0.005	0.296	0.378	0.008	0.008
6						0.003	0.003	0.575	0.230	0.048	0.005	0.066	0.625	0.002	0.002
7							1.000	0.003	0.003	0.048	0.078	0.008	0.008	0.741	0.851
8								0.005	0.005	0.005	0.078	0.008	0.008	0.899	0.546
9									0.315	0.403	0.043	0.037	0.784	0.006	0.004
10										0.006	0.006	0.032	0.296	0.004	0.004
11											0.055	0.006	0.006	0.004	0.004
12												0.006	0.006	0.004	0.004
13													0.043	0.004	0.004
14														0.004	0.004
15															0.378
